# Two cycles of adjuvant carboplatin for clinical stage 1 testicular seminoma in New Zealand centres: A retrospective analysis of efficacy and long‐term events

**DOI:** 10.1002/cnr2.1310

**Published:** 2020-10-26

**Authors:** Elias A. Chandran, Aaron Chindewere, Richard North, Michael B. Jameson

**Affiliations:** ^1^ Department of Oncology Waikato Hospital Hamilton New Zealand; ^2^ Department of Oncology Tauranga Hospital Tauranga New Zealand; ^3^ Waikato Clinical Campus University of Auckland Hamilton New Zealand; ^4^ Present address: Department of Medical Oncology Auckland City Hospital Auckland New Zealand; ^5^ Present address: Department of Oncology North West Regional Hospital Burnie, Tasmania Australia

**Keywords:** adjuvant chemotherapy, carboplatin, long‐term safety, seminoma, testicular cancer

## Abstract

**Background:**

Adjuvant carboplatin reduces relapse risk in clinical stage 1 (CS1) seminoma, though there is a paucity of long‐term safety data.

**Aim:**

Our objective was to report long‐term outcomes of two cycles of adjuvant carboplatin dosed at area under the time–concentration curve (AUC) of 7.

**Methods and results:**

We performed a retrospective analysis on treatment and outcomes of patients with CS1 seminoma who received adjuvant carboplatin from 2000 to 2016 at our centres in the Midland Region, New Zealand. Of 159 patients, median age 39 years, 153 received two cycles of carboplatin: 147 dosed at AUC7 and 6 at AUC6. Six patients had one cycle of carboplatin AUC7. One patient relapsed at 22 months and died of bleomycin pneumonitis 2 months after achieving a complete response with BEP chemotherapy. Neither RTI (present in 21.3%) nor tumor size >4 cm (in 43.3%) was predictive of relapse. Median follow‐up was 106 months. At 15 years, outcomes were: relapse‐free survival 99.4%, overall survival 91.4%, disease‐specific survival 100%, subsequent malignant neoplasm rate 7.6%, and second testicular germ cell tumor rate 3.85%. One patient had persistent grade 1 thrombocytopenia at 46 months.

**Conclusions:**

These data add to the body of evidence that two cycles of carboplatin AUC7 is safe and effective adjuvant treatment for CS1 seminoma.

## 1 INTRODUCTION

Seminoma accounts for more than half of testicular germ cell tumors (GCTs), with peak incidence at 35 to 45 years of age.^1^, ^2^ New Zealand Ministry of Health data from 2005 to 2017 show that Maori men have consistently higher rates of testicular cancer than non‐Maori men.^3^ About 80% of seminoma present with clinical stage 1 (CS1) disease, with an estimated relapse rate of 13% to 20% without adjuvant treatment.^1^, ^4^, ^5^ However, the high curability at relapse has led to ongoing debate about whether optimal postoperative management is adjuvant treatment or surveillance.^1^, ^4^, ^6^

Historically, adjuvant radiotherapy was given but was associated with increased incidence of subsequent malignant neoplasms (SMNs) and cardiovascular events.^7^, ^8^ When the MRC TE19 study showed noninferiority of a single dose of adjuvant carboplatin chemotherapy to radiotherapy, the use of adjuvant radiotherapy diminished.^7^, ^9^ Adjuvant carboplatin has been further explored in nonrandomized trials, using one or two cycles dosed at an area under the time–concentration curve (AUC) of 7 and effectively reduces relapse^6^, ^8^ without association with significant late toxicities or SMN.^10^, ^11^

Surveillance avoids treatment in the majority of patients and has largely become the preferred strategy.^4^, ^8^, ^12^ However, relapsed patients are exposed to the far greater toxicity of cisplatin‐based chemotherapy.^7^, ^13^, ^14^ There is no consensus on duration of surveillance, which can be up to 10 years, requiring up to 10 abdominal CT scans. This exposes patients to significant doses of radiation, raising concerns of long‐term SMN risk.^4^, ^8^, ^15^ From a psychological perspective, it is well known that patients with testicular cancer experience fear of relapse; however, it is unclear whether this is increased by surveillance.^16^, ^17^

Risk‐based management is proposed by some studies^18^, ^19^, ^20^, ^21^ and guidelines,^22^ reserving adjuvant carboplatin for patients with one or both of rete testis involvement (RTI) or tumor size more than 4 cm. However, significant heterogeneity in the predictive value of these risk factors questions the reliability of this approach.^23^, ^24^

Since 2000, the standard of care for patients with CS1 seminoma at the Waikato, Lakes and Bay of Plenty District Health Boards (DHB), New Zealand, has been to consider two cycles of adjuvant carboplatin AUC7, given 3 weeks apart. Our objectives were to analyze this cohort and determine relapse‐free survival (RFS), overall survival (OS), disease‐specific survival (DSS), cause‐specific survival (CSS), which includes deaths from seminoma and treatment, and rates of long‐term toxicity, SMN, and second GCT. We also wanted to observe the association of RTI and tumor size >4 cm with relapse.

## 2 METHODS

We retrospectively analyzed data of patients over 18 years old with CS1 seminoma who received adjuvant carboplatin from 2000 to 2016. Data were sourced from a proprietary database (Aesculapius) of Medical Oncology patients seen at the Waikato and Lakes DHBs, the Bay of Plenty DHB cancer database, and the New Zealand Health Information Service. This included age, ethnicity, disease stage, tumor size, RTI status, tumor marker levels pre chemotherapy, chemotherapy regimen including number of cycles intended and delivered, relapse, mortality, cause of death, and incidence of SMN (including contralateral GCT). Mortality and SMN data acquired from the national database were updated to December 12, 2017.

Descriptive statistics were used for patient, tumor, and treatment characteristics. Relapse according to RTI and tumor size >4 cm was analyzed using Fisher's exact test, and actuarial survival was estimated with the Kaplan–Meier method with asymmetrical 95% confidence interval (CI) recommended as more accurate than the more commonly used symmetrical confidence intervals by GraphPad Prism version 8.4.3 (GraphPad, CA, USA), which was used for all analyses. The study was conducted under approval from the Southern Health and Disability Ethics Committee (ref: 16/STH/251).

## 3 RESULTS

### 3.1 Patient characteristics

There were 159 patients with CS1 seminoma treated with adjuvant carboplatin. Three patients who developed a metachronous contralateral CS1 seminoma within the study period were treated with adjuvant carboplatin on both occasions and are counted twice.

Patient and disease characteristics are shown in Table 1. Median follow‐up for survival was 106 months (interquartile range 72‐159 months). Six patients had a prior history of testicular seminoma at a median of 7 (range 6‐10) years earlier, three of whom had received radiotherapy. Three patients had stage S1 due to raised LDH.

**TABLE 1:** Patient characteristics

Characteristic	N = 159	%
Age – median (range) years	39 (20‐73)	
Ethnicity		
New Zealand European	110	69.2
Maori	46	28.9
Other	3	1.9
AJCC staging (seventh edition)		
T1	130	81.8
T2	23	14.5
T3	6	3.8
N0	159	100.0
N1	0	0.0
S0	139	87.4
S1	3	1.9
Sx	16	10.1
Tumor size		
>4 cm	69	43.3
<4 cm	78	49.1
Not known	12	7.5
Rete testis invasion		
Yes	34	21.4
No	72	45.3
Not known	53	33.3

*Note*: Sx: serum tumor marker status unknown.

### 3.2 Treatment

One hundred forty‐seven of 153 patients (96%) received their planned two cycles of carboplatin AUC7. Six patients received one cycle: one patient by intention, three due to adverse effects (one each of nausea and vomiting, neuropathy, and hypersensitivity reaction), one due to attempted suicide and one due to incarceration. Six patients received two cycles of carboplatin AUC6, one due to chronic kidney disease; the other five had no documented reason for this dose. Glomerular filtration rate was largely estimated by the Cockcroft‐Gault equation; however, in patients at extremes of body habitus, it was measured by ^51^Cr‐EDTA clearance.

Acute toxicity was not systematically recorded, but there were only two acute admissions during treatment: one with nausea and vomiting and the other with headache. Persistent adverse effects were rare: there was one case of ongoing grade 1 thrombocytopenia 46 months post chemotherapy.

### 3.3 Follow‐up

After completing chemotherapy, patients had clinical examinations and tumor markers checked every 3 to 6 months for the first 2 years and up to 5 years depending on clinician preference. Most patients only had one CT scan at month 12, but, depending on estimated risk of relapse, some had up to four CT scans over the first 5 years. Fifteen (9.4%) patients were lost to follow‐up due to noncompliance.

### 3.4 Outcomes

**FIGURE 1:**
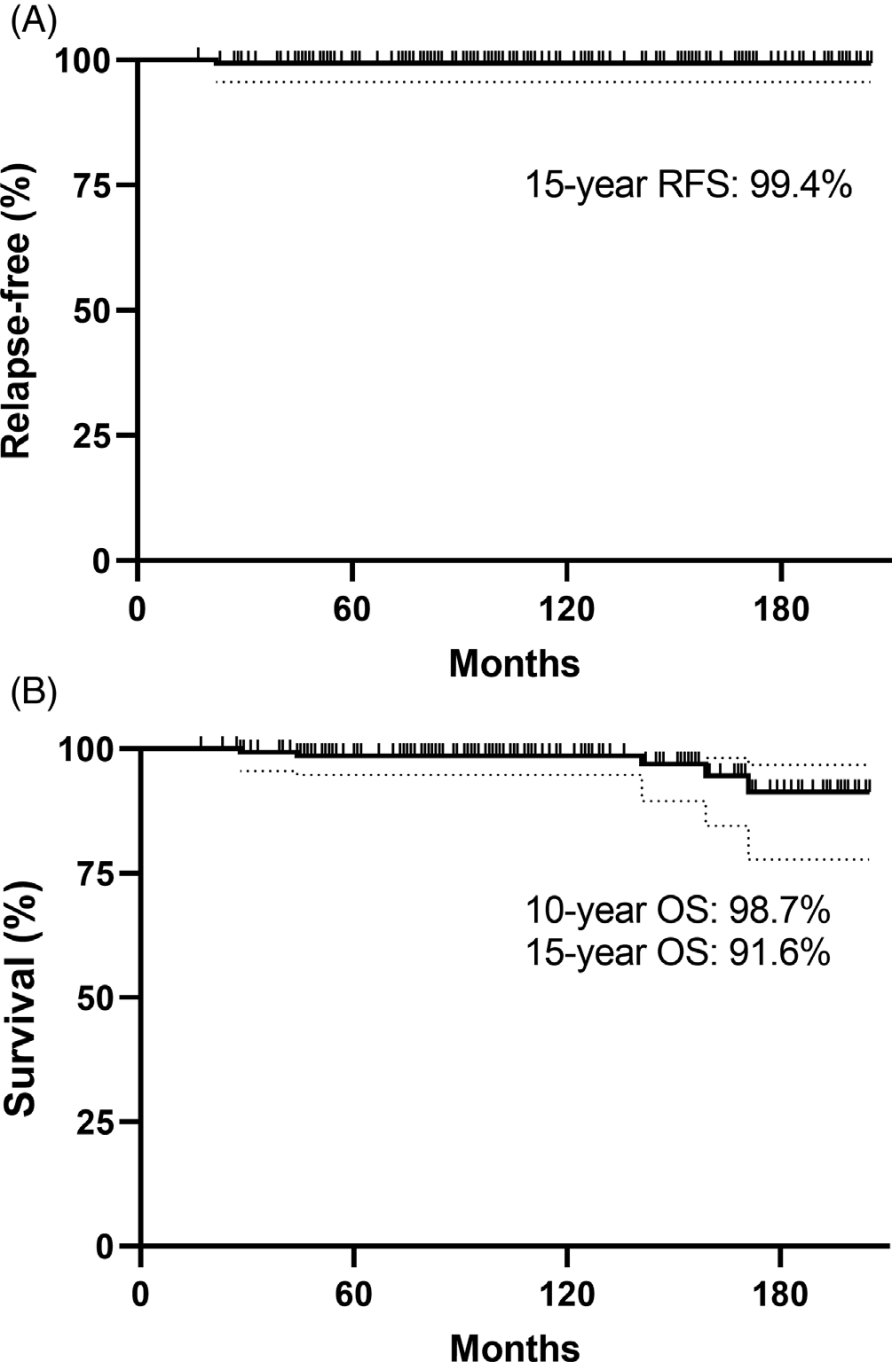
Survival. A: relapse‐free survival; B: overall survival. Dotted lines represent asymmetrical 95% CI

One patient aged 47 years at initial diagnosis, of NZ European descent, relapsed in his para‐aortic nodes at 22 months following two cycles of carboplatin dosed at AUC7, resulting in actuarial RFS of 99.4% (95% CI 95.6‐100) at 15 years (Figure 1A). He achieved a radiological complete response after four cycles of BEP but unfortunately died 2 months later of bleomycin pneumonitis precipitated by a large pulmonary embolus requiring high‐flow oxygen. Including the relapsed patient, there were five deaths, the remaining four due to SMN (Table 2), of whom one was Maori. No patients died from progressive seminoma. OS was 98.7% (95% CI 97.7‐100) and 91.4% (95% CI 85.9‐100) at 10 and 15 years, respectively (Figure 1B). DSS and CSS at 15 years were 100% and 99.4%, respectively.

**TABLE 2:** Subsequent Malignant Neoplasms

SMN	Age at SMN diagnosis	Time from chemotherapy (months)	Died of SMN
Second germ cell tumors			
Contralateral CS1 seminoma	36	80	No
Contralateral CS1 seminoma	37	48	No
Contralateral CS1 seminoma	41	58	No
Contralateral CS1 seminoma	41	126	No
Other SMNs			
Neuroendocrine carcinoma of axilla	44	36	No
Melanoma	48	130	Yes
Glioblastoma multiforme	51	38	Yes
Rectal adenocarcinoma	51	51	No
Myeloma	56	96	Yes
Small cell lung cancer	64	132	Yes
Prostate adenocarcinoma	69	102	No

Abbreviation: CS1: clinical stage 1.

RTI status was reported in 106 patients (Table 1): 21 patients (13.2%) had both RTI and tumor size >4 cm. The relapsed patient had both risk factors. However, neither RTI nor tumor size >4 cm significantly affected the relapse rate (*P* = .32 and .47, respectively).

### 3.5 Subsequent malignant neoplasms

Eleven SMNs occurred, four of which were contralateral seminomas (Table 2). Actuarial second GCT incidence at 15 years was 3.85% (95% CI 0‐30.1). Seven non‐GCT SMNs were diagnosed at a median of 96 months post chemotherapy, with actuarial incidence 7.6% at 15 years (95% CI 0.3‐31.3). None occurred in patients who previously received radiotherapy for prior GCT. Median age at diagnosis of second GCT and SMN was 39 and 51 years, respectively.

## 4 DISCUSSION

The 15‐year RFS of 99.4%, OS of 91.4%, and DSS of 100% in our population provides further evidence for the efficacy of two cycles of adjuvant carboplatin for CS1 seminoma. The ideal number of cycles of carboplatin has not been defined in a randomized controlled trial (RCT), but nonrandomized studies and interstudy comparison suggest inferiority of one cycle compared to two, summarized in Table 3. Relapse rates were 0% to 8.6% vs 0% to 3.3% for one vs two cycles of carboplatin, respectively, though there was considerable heterogeneity of follow‐up duration and study populations (Table 3). The absence of an adequately powered RCT is likely due to the requirement for about 5000 patients to detect superiority of two cycles vs one cycle of adjuvant carboplatin.

**TABLE 3:** Studies of CS1 seminoma treated with adjuvant carboplatin (dosed at AUC7 unless stated otherwise)

Author	Type of study	Cycles of carboplatin	Patients (N)	Population	Median F/U (months)	Relapse Rate (%)/time	Second GCT rate (%)	SMN % (site/*months*)	DFS (%)	5y DSS (%)	5y OS (%)
Carboplatin 1 cycle											
Oliver 2011^9^	RCT	1	573	pT1‐3, normal post‐op HCG	78	5.1 (*NS*)	0.3 (*NS*)	0.9 unspecified (*NS*)	94.7 (RFR)	100	99
Tandstad 2011^7^	Prospective, non‐randomised	1	188	All comers (T > 4 cm 52.7%)	41	3.9 (*0.9 ‐32 m*)	NS	NS	NS	100	99.2
Carboplatin 2 cycles											
Aparicio 2018^20^	Prospective non‐randomised	2	64	RTI	33	1.6 (*20 m*)	NS	NS	98.2 at 3y	100 at 3y	100 at 3y
Aparicio 2011^29^	Prospective, non‐randomized	2	74	RTI and T > 4 cm	74	1.4 (*25 m*)	NS	NS	88.1 at 3y	100 at 3y	100 at 3y
Steiner 2010^30^	Retrospective	2 cycles 400 mg/m^2^	282	All comers (T > 4 cm 48.2%, RTI NS)	75.2 (mean)	1.06 (*9‐22 m*)	1.9 (*2‐10.8y*)	1.8 (2 prostate, 2 melanoma, 1 RCC/*NS*)	98.1	100	NS
Argirovic 2009^31^	Prospective	2 cycles 400 mg/m^2^	230	All comers	84	2.6 (*median 31 m*)	1.7 (*median 20.3 m*)	0.4 (Lung/*28 m*)	NS	100 at 7y	99.1 at 7y
Aparicio 2005^32^	Prospective, non‐randomized	2	214	RTI 38.8%, T > 4 cm 84.6%, both 23.4%	34	3.3 (*4‐28 m*)	0.9 (*NS*)	0.9 (1 RCC, 1 CLL/*NS*)	96.2	100	100
Aparicio 2003^33^	Prospective, non‐randomized	2 cycles 400 mg/m^2^	60	T2 or venous/lymphatic vascular invasion	52	3.3 (*median 11 m*)	NS	0%	96.6	100	NS
Reiter 2001^10^	Prospective, non‐randomized	2 cycles 400 mg/m^2^	107	All comers	74	0	0	0.9 (rectal/*26 m*)	100 at 74 m	100 at 74 m	94.4 at 74 m
Krege 1997^34^	Phase 2 single arm	2 cycles 400 mg/m^2^	43	All comers	28	0	NS	NS	NS	NS	NS
Carboplatin varying number of cycles or not stated											
Ruf 2019^5^	Retrospective	1	161	All comers	96	NS	NS	5 (ALL/*2*, prostate/*10‐210*, CUP/*16*, melanoma/*19‐97*, NET/*34*, MGUS/74, RCC/*111*, Pancreas/*164)*	NS	100	NS
		2	82							100	
Tyrrell 2017^21^	Prospective, non‐randomized	NS	175	All comers	NS	6.2 (*NS*)	NS	NS	NS	NS	NS
Diminutto 2016^35^	Retrospective	1	107	CS1 seminoma, normal post‐op HCG RTI 28.7% T > 4 cm 17.4% Both 35.7%	22.1	5.2 (*11.1‐16.6 m*)	0.9 (*27 m*)	0.9 (multiple myeloma in patient with pre‐existing MGUS/*47.4*)	94.8 PFS at 2y	99.5 at 2y	99.5 at 2y
		2	8								
Dieckmann 2016^36^	Prospective, non‐randomized	1	362	All comers	30	5 (*NS*)	NS	NS	NS	100	NS
		2	66		30	1.5 *(NS)*				100	
Glaser 2015^37^	Retrospective	NS	3508	All comers	67	NS	NS	NS	NS	NS	97.7
Powles 2008^11^	Prospective, non‐randomized	1	28	All comers (RTI 24%, T > 4 cm 47%, both 11.1%)	108	2 (24*‐72 m*)	2.5 (*5.8‐11.4y*)	2 (SCLC, meningioma, Hodgkin, Prostate/*6.7‐13.7y*)	NS	100 at 9y	96.5 at 9y
		2	171								
Oliver 2001^38^	Phase 2 non‐randomized and randomized	1	146	All comers	52	0.7 (NS)	0.7	0	100	100	100
		2	57		128	1.75 (NS)	0	3.5	96.5	100	96.5
Dieckmann 2000^39^	Prospective, non‐randomized	1 cycle 400 mg/m^2^	93	All comers	48	8.6 (*median 16 m*)	1.1 (*4y*)	NS	91.1	100	100
		2 cycles 400 mg/m^2^	32			0	0	3.1 (NPC*/4y*)	NS	100	100
Oliver 1994^40^	Prospective non‐randomized	1	25	All comers	29	0	0	NS	99	NS	NS
		2 (cisplatin n = 3)	53		51	1					

Abbreviations: ALL, acute lymphoblastic leukaemia; AUC, area under the time concentration curve; CLL, chronic lymphocytic leukaemia; CS1, clinical stage I; CUP, carcinoma of unknown primary; GCT, germ cell tumor; m, month; MGUS, monoclonal gammopathy of uncertain significance; NPC, nasopharyngeal carcinoma; NS, Not stated; PFS, progression‐free survival; RCC, renal cell carcinoma; RFR, relapse‐free rate; RTI, rete testis involvement; SCLC, small cell lung cancer; SMN, subsequent malignant neoplasm; T, tumor; y, year.

Controversy remains about the predictive value of tumor size >4 cm and RTI for relapse.^24^ They were not predictive of relapse in our study.

While we did not prospectively record adverse events in our study, others report relatively mild toxicity with carboplatin, excellent treatment completion rates, and no excess in overall mortality or death from cardiovascular disease.^10^, ^11^ A recent study by Ruf et al with median follow‐up of 142 months reported a 13.2% hypogonadism rate but no major impact on fertility among 234 patients who had received one or two cycles of carboplatin.^5^

There has been a general shift toward surveillance to minimize treatment burden in CS1 seminoma.^4^, ^8^, ^12^ A 2015 meta‐analysis including 12 075 patients from 13 trials found no OS benefit of chemotherapy or radiotherapy over surveillance despite an 80% reduction in relapse, justifying the role for surveillance.^6^ However surveillance requires excellent compliance with frequent clinical reviews and investigations for up to 10 years.^8^ Radiation from CT scanning increases the SMN risk by 1 in 1000 per 10mSV, with each abdominopelvic CT scan equivalent to 10 to 20mSV.^4^, ^8^, ^15^ Non‐compliance with surveillance was only 4.7% in a large Danish study; however, patients who default surveillance may compromise their chances of cure.^12^ While the relapse risk after adjuvant chemotherapy is much lower, it is still concerning that 9.3% of our patients were noncompliant with recommended follow‐up.

Relapsed patients are mostly treated with BEP chemotherapy, which has much greater acute and late toxicity than carboplatin, including hearing loss, tinnitus, neurotoxicity, nephrotoxicity, gonadal toxicity, increased cardiovascular risk, and possibly SMN.^25^, ^26^, ^27^ Our relapsed patient and one of 69 relapsed patients in SWENOTECA VII died of BEP‐related complications.^19^ However, no significant difference in noncancer mortality between surveillance and adjuvant carboplatin treatment has been found.^6^

In frail or older patients with CS1 seminoma who may be poor candidates for cisplatin‐based chemotherapy, the significant lowering of relapse risk with adjuvant carboplatin may be desirable.

Our second GCT rate of 3.85% at 15 years appears higher than in other studies (0.54%‐2.5%, Table 3), though the 95% CI includes zero, and our follow‐up is longer than in some of these studies. While the TE19 trial suggested that carboplatin reduced the second GCT rate, perhaps due to effects of in‐situ neoplasia in the contralateral testis, second GCT rates in other carboplatin groups have been similar to surveillance.^11^, ^18^

The 15‐year non‐GCT SMN rate of 7.6% also appears higher than in other studies (0.9‐5%),^5^, ^27^ although the 95% CI includes zero, and there are differences in follow‐up duration. Prospective studies have reported similar SMN rates between patients treated with adjuvant carboplatin compared with surveillance or the general population.^11^, ^18^ Our rates of prostate cancer and melanoma (both 0.6%) are lower than those reported by Ruf et al,^5^ who noted higher‐than‐expected incidence (both 1.2%) among patients who had adjuvant treatment. It is likely that the SMN rates reported in our smaller sample size are not significantly different to the other studies.

Despite the national incidence of testicular cancer being higher among Maori, the proportion of Maori men in our study (28.9%) was similar to regional demographic data.^28^ Similarly, there was no difference in actuarial survival between Maori and non‐Maori patients (log‐rank *P* = .854).

We acknowledge as limitations the retrospective nature of our study, lack of standardized reporting on tumor size and RTI, lack of long‐term data on infertility, hypogonadism and cardiovascular disease, and the relatively small sample size.

## 5 CONCLUSION

Our findings further support the efficacy of two cycles of adjuvant carboplatin AUC7 for CS1 seminoma and demonstrate its long‐term safety, comparable with other published studies.

## CONFLICT OF INTEREST

Elias A. Chandran received the ANZUP/AstraZeneca Travel Fellowship 2019. The authors make no other declarations.

## AUTHORS' CONTRIBUTIONS

All authors had full access to the data in the study and take responsibility for the integrity of the data and the accuracy of the data analysis. *Conceptualization*, A.C., R.N., M.B.J.; *Data curation*, E.A.C., A.C., R.N., *Project administration*, E.A.C., R.N., M.B.J.; *Methodology*, A.C., M.B.J.; *Investigation*, M.B.J.; *Formal Analysis*, E.A.C., M.B.J.; *Writing ‐ Original Draft*, E.A.C.; *Writing ‐ Review & Editing*, E.A.C., M.B.J.; *Supervision*, M.B.J.

## ETHICAL STATEMENT

Data collection and analysis for this study was approved by the Southern Health and Disability Ethics Committee (ref: 16/STH/251). Patient consent statment was not applicable.

## Data Availability

De‐identified raw data from this study will be available on request
